# Modified single-patch technique: Repairing complete atrioventricular septal defect

**DOI:** 10.4103/0974-2069.52808

**Published:** 2009

**Authors:** Carl Lewis Backer, Sunjay Kaushal, Constantine Mavroudis

**Affiliations:** Division of Cardiovascular-Thoracic Surgery, Children's Memorial Hospital, Feinberg School of Medicine, Northwestern University, Chicago, IL, USA; 1 Ross Professor of Surgery, Cleveland Clinic Foundation, 9500 Euclid Avenue, M41 - 02 Cleveland, OH, USA

## INTRODUCTION

The modified single-patch technique was conceived simultaneously by two separate groups, one in the United States and one in Australia. Dr. Benson Wilcox along with Dr. Robert Anderson, reported in 1997, a technique where 12 patients with complete atrioventricular (AV) septal defect had direct closure of the ventricular element.[1] This technique resulted in significantly shorter cardiopulmonary bypass and aortic cross-clamp times. The intermediate term follow-up suggested that the results were comparable to those of a more standard two-patch technique. In 1999, Dr. Graham Nunn from Australia reported on 47 consecutive patients who were operated on between 1995 and 1998.[2] In all these patients, repair was performed by direct suturing of the common atrioventricular valve leaflet to the crest of the ventricular septum. Nunn concluded that this technique greatly simplified the repair and did not lead to left ventricular outflow tract obstruction or interfere with valve function.

The results reported by these institutions led us, over a period of six years, to evolve from our reliance on the classic two-patch technique to a modified single-patch technique during the years 2000-2006.[3] The name of this technique has evolved from the “simplified approach” to “simplified single-patch” technique to “modified single-patch” technique.

## TECHNICAL DETAILS

**Figure 1:**
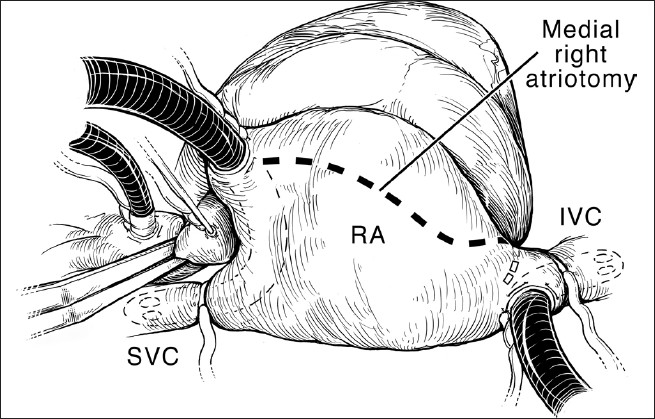
Atrial incision. Aortic and bicaval venous cannulation has been accomplished. The aorta has been cross-clamped, and cold blood cardioplegia is being administered. The caval tapes have been snared. The dotted line indicates the medial right atrial incision. This incision extends from the right atrial appendage, parallel to the right AV groove and the right coronary artery. Note that the most inferior extent of the incision is between the inferior vena cava (IVC) and the right coronary artery and coronary sinus. This incision allows excellent mobilization of the ventricle and resultant exposure of the AV valves. RA, right atrium; SVC, superior vena cava. (Reprinted with permission: Backer CL, Mavroudis C. Atrioventricular Canal Defects. In: Mavroudis C, Backer CL, [eds]: Pediatric Cardiac Surgery, 3^rd^ ed., Philadelphia, 2003, Mosby)

**Figure 2:**
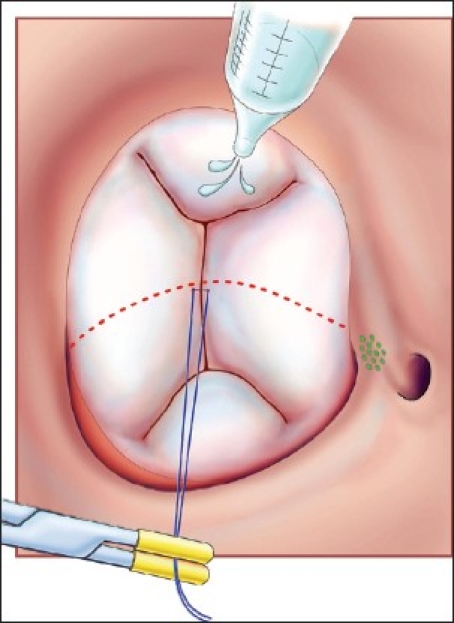
The common AV valve is “floated” with cold saline to identify and mark the apex of the zone of apposition with a 6.0 or 7.0 prolene suture. This also marks the dividing point between the projected right and left AV valves. (Reprinted with permission: Backer CL, Stewart RD, Mavroudis C. Semin Thorac Cardiovasc Surg 2007;19:249-257)

**Figure 3:**
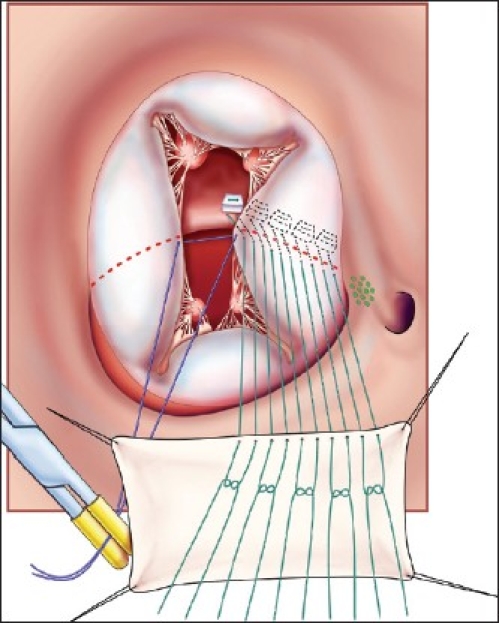
Pledgeted sutures (5-0 Ticron) are placed on the right ventricular side of the crest of the ventricular septal component of the AV septal defect. They are then sequentially passed through first the midportion of the common AV valve and then through an autologous pericardial patch. The location of the AV node is indicated by the dotted green oval. (Reprinted with permission: Backer CL, Stewart RD, Mavroudis C. Semin Thorac Cardiovasc Surg 2007;19:249-257).

**Figure 4:**
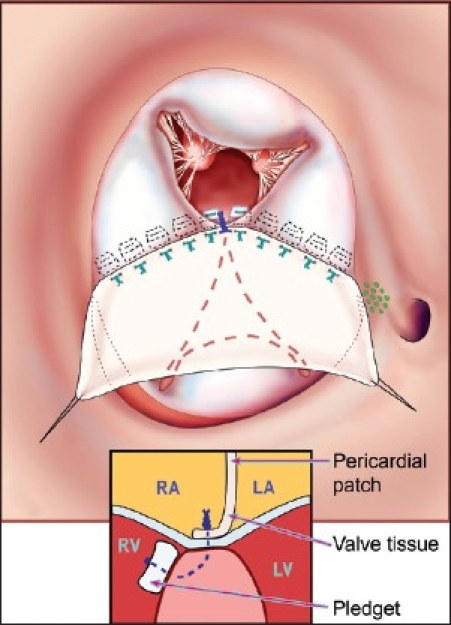
The pledgeted sutures have now sandwiched the AV valve tissue between the ventricular endocardium and the harvested pericardial patch. This effectively septates the common AV valve into a right-sided AV valve and a left-sided AV valve. The left-sided AV valve is shown in the dotted lines under the pericardial patch. The right AV valve is anterior. The small inset shows how the pledget and suture sandwich the ventricular endocardium, AV valve, and pericardium. (Reprinted with permission: Backer CL, Stewart RD, Mavroudis C. Semin Thorac Cardiovasc Surg 2007;19:249-257).

**Figure 5:**
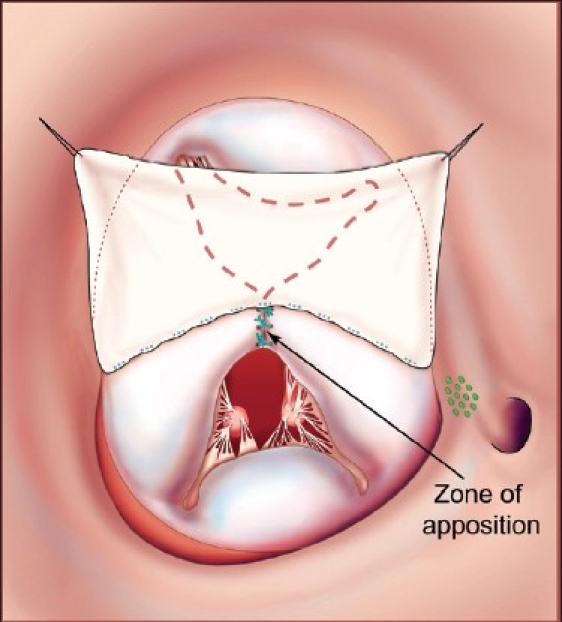
Interrupted prolene sutures are used to approximate the zone of apposition and ensure that the left-sided AV valve is competent and does not develop late insufficiency. (Reprinted with permission: Backer CL, Stewart RD, Mavroudis C. Semin Thorac Cardiovasc Surg 2007;19:249-257)

This operation is performed with cardiopulmonary bypass, moderate hypothermia (28°C), and aortic cross-clamp, with cold blood cardioplegia. After sternotomy and establishing cardiopulmonary bypass with bicaval venous cannulation, a vent is placed in the right superior pulmonary vein. The operation is facilitated by direct cannulation of the superior vena cava with a right-angle cannula. After administration of cold blood cardioplegia a medial right atriotomy is performed. This incision is very important and extends between the inferior vena cava cannula and the right coronary artery [Figure 1]. This allows the atrium to be opened very wide and gives maximum exposure of the common AV valve. The vent is withdrawn into the left atrium and the first part of the repair utilizes a bulb syringe to inject saline into the common AV valve [Figure 2]. By floating the common AV valve, the location of the projected midportion of the common AV valve where it will be separated into right and left components is established. We mark this “zone of apposition” point with a 6.0 or 7.0 prolene suture. This becomes the dividing point between the projected right and left AV valve. Next, a series of pledgeted sutures are placed on the right ventricular side of the crest of the ventricular septum [Figure 3]. These sutures are passed through the projected midpoint of the dividing line between the common AV valve leaflets. One of the reasons this operation is successful is because the superior and inferior bridging leaflets are not divided. After passing the suture through the AV valve tissue it is passed through a previously harvested autologous pericardial patch [Figure 3]. The sutures are tied and cut and this obliterates the ventricular component of the AV septal defect. The pledget in the myocardium and the pericardium buttress the apposition of the valve tissue to the ventricular septum. This helps prevent valve dehiscence during the postoperative period [Figure 4]. Next, the pericardial patch is reflected anteriorly and the zone of apposition of the left AV valve is approximated [Figure 5].

**Figure 6:**
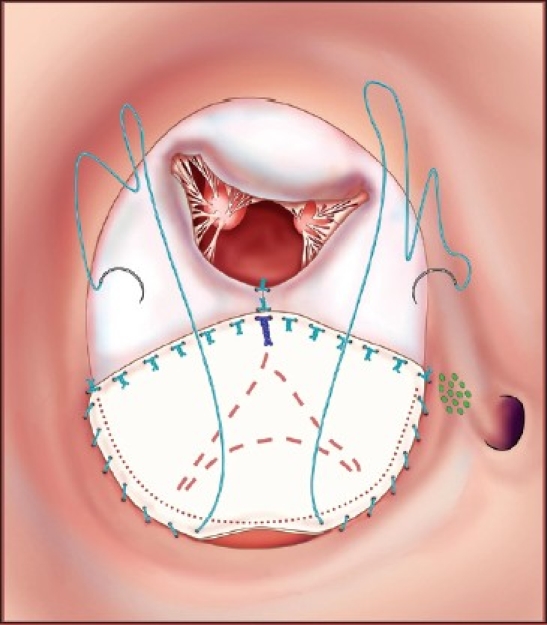
Running prolene suture is used to approximate the pericardium to the edges of the atrial septal defect effectively closing the atrial component of the AV septal defect. The zone of apposition of the right-sided AV valve is usually approximated with only two or three interrupted prolene sutures. The coronary sinus is kept draining to the right atrium. (Reprinted with permission: Backer CL, Stewart RD, Mavroudis C. Semin Thorac Cardiovasc Surg 2007;19:249-257)

We used interrupted simple prolene sutures for this approximation. We continued placing sutures until the point where the chordae insert on the AV valve leaflet, both the superior and inferior bridging leaflets. Finally, the pericardial patch was used to close the atrial component of the AV septal defect [Figure 6]. The pericardial patch was sutured in place with running 5.0 or 6.0 polypropylene sutures. The location of the AV node is illustrated by the green dots in this series of illustrations. The AV node was avoided by placing the suture line very close to the inferior bridging leaflet and not bringing the suture line up to the atrial septum till it passed the coronary sinus. The right atrium was closed with two layers of running prolene sutures and the right and left sides of the heart were deaired in the usual fashion. The patients were usually weaned from cardiopulmonary bypass on 0.5 mcg/kg/minute of milrinone along with low-dose dopamine and/or dobutamine. We performed modified ultrafiltration on all patients for 10 minutes. The patients were evaluated by postoperative transesophageal echocardiography during the time of modified ultrafiltration.

**Figure 7a and b:**
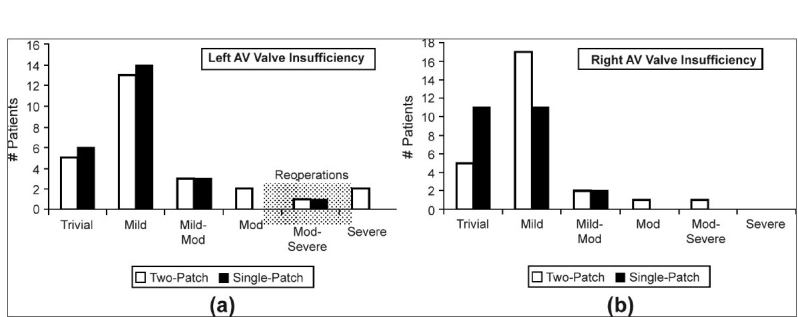
Degree of left (a) and right (b) atrioventricular (AV) valve insufficiency as assessed by postoperative transthoracic echocardiography. The AV valve insufficiency was graded as trivial, mild, mild-to-moderate, moderate, moderate-to-severe, and severe along the x axis. The y axis indicates the number of patients in the two different groups (single-patch and two-patch). (Reprinted with permission: Backer CL, Stewart RD, Mavroudis C. Semin Thorac Cardiovasc Surg 2007;19:249-257)

We compared the results of the modified single-patch technique with the two-patch technique at our institution between 2000 and 2006.[3] There was no difference in postoperative mortality, with one death in the modified single-patch group (postoperative day 130, liver failure) versus no deaths in the two-patch group. Cross-clamp times and cardiopulmonary bypass times were shorter in the modified single-patch group (97.3 ± 19.9 versus 123.3 ± 28.2 minutes, *P* < 0.0003: 128 ± 25 versus 157 ±37, *P* < 0.03). Rastelli classification was similar; type A was 18 versus 14, type B, 1 versus 0, and type C, 7 versus 15. The mean size of the ventricular septal defect as assessed by transesophageal echocardiography was 9 ± 2 mm in the single-patch group versus 10 ± 3 mm in the two-patch group. There was no difference in the median postoperative length of stay (10 versus 8 days). The AV valve insufficiency was evaluated as trivial, mild, moderate, and severe. There was no difference in the degree of postoperative left or right AV valve insufficiency as assessed by serial echocardiography [Figure 7a and b]. There was one reoperation for left AV valve insufficiency in the modified single-patch group versus three in the two-patch group (4% versus 10%, *P* = ns). There were no patients with third degree AV block or requirement for reoperation for residual ventricular septal defect (VSD) in the modified single-patch group. There was one patient with third degree AV block, who required a pacemaker, and one patient that had a residual VSD in the two-patch group. No patient in either group required reoperation for left ventricular outflow tract obstruction. From our comparison, the modified single-patch technique produced results comparable with the two-patch technique in younger patients with similar sized ventricular septal defects. The mean age at repair in the single-patch group was 4.4 ± 1.3 months. The mean weight was 4.74 ± 0.92 kg. The modified single-patch technique was performed with significantly shorter cross-clamp and cardiopulmonary bypass times.

## DISCUSSION

I was recently asked to write an invited commentary on the superb results from the Michigan group, on patients with complete AV septal defects.[4] On analyzing those results and comparing them with the modified single-patch technique, ther were two aspects of the modified single-patch technique, on meta-analysis, which appeared to be an improvement on the classic two-patch technique. In a combined series of 200 patients undergoing the modified single- patch technique from the Children's Memorial group, Dr. Richard Jonas' experience[5] and Dr. Graham Nunn's[6] most recent publication showed that the incidence of left AV valve reoperation was only 2%. This compared with an incidence of left AV valve reoperation in the two-patch technique, which was 7% of 889 patients, and the classic one-patch technique, which was 9.7% of 350 patients.

It is my strong impression that the modified single-patch technique may be a technically superior surgical approach that will improve left AV valve function. By operating on younger patients (three to four months of age), by not dividing the AV valve leaflets, and by not placing a patch for the VSD we may be able to decrease the frequency of reoperations on the left AV valve.

One other outcome measure that has been raised as a concern for the modified single-patch technique is left ventricular outflow tract obstruction. If one looks at the data, the incidence of left ventricular outflow tract obstruction requiring reoperation in the recent series from the University of Michigan (mostly two-patch technique) was 5%[4] Among a collected series of 200 patients undergoing the modified single-patch technique, some patients who were operated on by Dr. Nunn, now over 10–15 years (n = 26), there were no patients who required reoperation for left ventricular outflow tract obstruction.[6] Although it may be counterintuitive, in actuality the synthetic patch placed in a two-patch technique may actually promote fibrosis that leads to obstruction of the left ventricular outflow tract because of increased rigidity, turbulence of blood flow, and the presence of a foreign body in the left ventricular outflow tract.

An advantage of the modified single patch technique over the two-patch technique with regard to patch placement is the actual creation of the patch. For surgeons with less experience, the sizing and shape of the patch placed to close the ventricular septal defect can be difficult. This patch if too large will adversely affect the height of the AV valve and may lead to AV valve insufficiency. If the patch is too small it may not be sufficient, given that the strategy is to close the actual ventricular component. One of the reasons that we progressed to the modified single-patch technique is that we found our results seemed to improve as we made the VSD patch smaller. At a certain point it seemed logical to avoid the patch altogether.

We have not used this technique for patients with tetralogy of Fallot and atrioventricular septal defect, or patients with double outlet right ventricle with AV septal defect. In those patients the ventricular component is significantly larger and has required a patch to provide unobstructed flow from the left ventricle to the aorta. However, a slight modification of our technique for these patients has been to use the modified single-patch technique for the inferior bridging leaflet. The inferior bridging leaflet is attached directly to the crest of the ventricular septum as in the standard modified single-patch technique. A Gore-tex patch is then placed underneath the superior bridging leaflet to direct the left ventricular blood to the aorta, which is overriding in patients with tetralogy and originating solely from the right ventricle in patients with double outlet right ventricle.

## CONCLUSION

In conclusion it is our strong belief that the modified single-patch technique is now the best technique available for the repair in patients with complete atrioventricular septal defect. This approach is associated with very low operative mortality, it is easily applied to small infants, and the incidence of reoperation for left AV valve insufficiency and left ventricular outflow tract obstruction is significantly lower than previously reported, for either the classic single-patch technique or the two-patch technique.
